# Evaluation of the lower protein limit in the budding yeast *Saccharomyces cerevisiae* using TIPI-gTOW

**DOI:** 10.1186/1752-0509-8-2

**Published:** 2014-01-07

**Authors:** Masataka Sasabe, Sayumi Shintani, Reiko Kintaka, Kazunari Kaizu, Koji makanae, Hisao Moriya

**Affiliations:** 1 Research Core for Interdisciplinary Sciences, Okayama University, Kita-ku, Okayama, Japan; 2 Graduate School of Natural Science and Technology, Okayama University, Kita-ku, Okayama, Japan; 3 Quantitative Biology Center, RIKEN, Suita, Osaka, JAPAN

## Abstract

**Background:**

Identifying permissible limits of intracellular parameters such as protein expression provides important information for examining robustness. In this study, we used the TEV protease-mediated induction of protein instability (TIPI) in combination with the genetic Tug-of-War (gTOW) to develop a method to measure the lower limit of protein level. We first tested the feasibility of this method using *ADE2* as a marker and then analyzed some cell cycle regulators to reveal genetic interactions.

**Results:**

Using TIPI-gTOW, we successfully constructed a strain in which GFP-^TDegF^Ade2 was expressed at the lower limit, just sufficient to support cellular growth under the -Ade condition by accelerating degradation by TEV protease. We also succeeded in constructing a strain in which the minimal level of GFP-^TDegF^Cdc20 was expressed by TIPI-gTOW. Using this strain, we studied genetic interactions between cell cycle regulators and *CDC20*, and the result was highly consistent with the previously identified interactions. Comparison of the experimental data with predictions of a mathematical model revealed some interactions that were not implemented into the current model.

**Conclusions:**

TIPI-gTOW is useful for estimating changes in the lower limit of a protein under different conditions, such as different genetic backgrounds and environments. TIPI-gTOW is also useful for analyzing genetic interactions of essential genes whose deletion mutants cannot be obtained.

## Background

Effective functioning of cellular systems requires optimal expression of individual proteins [1-3]. On the other hand, cellular systems are generally robust against changes in protein expression [4-6]. Identifying permissible limits of intracellular parameters such as protein expression provides important information for examining robustness [4-6].

We previously developed a method designated genetic Tug-Of-War (gTOW), by which we can measure the limit of overexpression of a target protein [7,8]. In gTOW, we clone a target gene with its native promoter into a 2 μ-based plasmid harboring *leu2d*. Under leucine-lacking (-Leu) conditions, the copy number of the plasmid reaches >100 copies because of the selection bias driven by *leu2d*. If the target protein expression reaches the upper limit, i.e., essential cellular functions come to a halt when the level of protein exceeds a certain limit, the gene/plasmid copy number must decrease to below the upper limit. In gTOW, we thus increase the expression of a protein to a limit by increasing the copy number and then determine the upper limit by measuring the corresponding copy number of the gene. Using gTOW, we previously measured the copy number limits for overexpression of 30 cell cycle regulatory genes in the budding yeast *Saccharomyces cerevisiae* and studied the robustness of the cell cycle and mechanisms that can improve the robustness of the cell cycle [7,9,10]. We recently measured the copy number limits of all yeast genes and showed that the yeast cellular system is robust against >100-fold increase in the copy numbers of each of >80% genes [11]. In that analysis, we also identified 115 “dosage-sensitive genes” whose copy number limits were <10 [11].

While gTOW is a method to measure the upper limit of protein expression, determination of the lower limit is still a question. The commonly used gene-deletion experiments reduce the expression of a target protein to 0. Gene-deletion experiments for all genes in *S. cerevisiae* have revealed that about 20% genes are essential for cellular growth under normal conditions [12]; other genes are essential for cellular growth under specific conditions such as specific environmental conditions or in combination with other gene knockouts [13,14]. Each of these proteins must have a minimal requirement level (i.e., the lower limit) to support cellular growth under each of these conditions. At present, there is no effective method to assess the lower expression limit of a target protein.

To assess the lower limit, we have to gradually reduce the expression of a protein and then measure the lower limit by some methods. To reduce the expression of a gene/protein, we need to reduce the production rate of the transcript, increase the degradation rate of the transcript, decrease the production rate of the protein, or increase the degradation rate of the protein. To identify the lower limit of expression, we would need to specifically decrease/increase the production/degradation rate of the target and evaluate the limit that would halt cellular function. Experimental methods that could potentially be used for this purpose in *S. cerevisiae* are as follows: (1) decreasing the expression rate of the transcript using a repressible promoter [15], (2) decreasing the transcription rate or increasing the degradation rate using RNA interference (RNAi) [16], (3) increasing the degradation of mRNA by the decreased abundance by mRNA perturbation (DAmP) method [17,18], (4) increasing the rate of degradation of the transcript by recruiting the RNA-degrading enzyme to the 3′ region of the transcript [19], (5) increasing the rate of degradation of the protein using inducible degrons [20,21], (6) increasing the rate of degradation of the protein using the TEV protease-mediated induction of protein instability (TIPI) method [22,23].

In this study, we used TIPI in combination with gTOW to develop a method to measure the lower limit of protein expression. In TIPI, a target protein is expressed with a TEV protease-induced degron, an N-terminal sequence containing a cleavage site for the site-specific TEV protease. Cleavage of the protein at this site by the TEV protease triggers the exposure of the “N-degron” [24], which triggers the rapid degradation of the target protein through the N-end rule pathway. We chose TIPI because, theoretically, we can increase the degradation rate of the target protein by increasing the expression of TEV protease using the gTOW scheme with a plasmid encoding TEV. We can then evaluate the lower limit of the target protein expression by measuring the copy number of the TEV plasmid. We first tested the feasibility of this method using *ADE2* as a marker and then analyzed some cell cycle regulators to reveal genetic interactions.

## Results and discussion

### Establishment of TIPI-gTOW using *ADE2* as a marker

**Figure 1:**
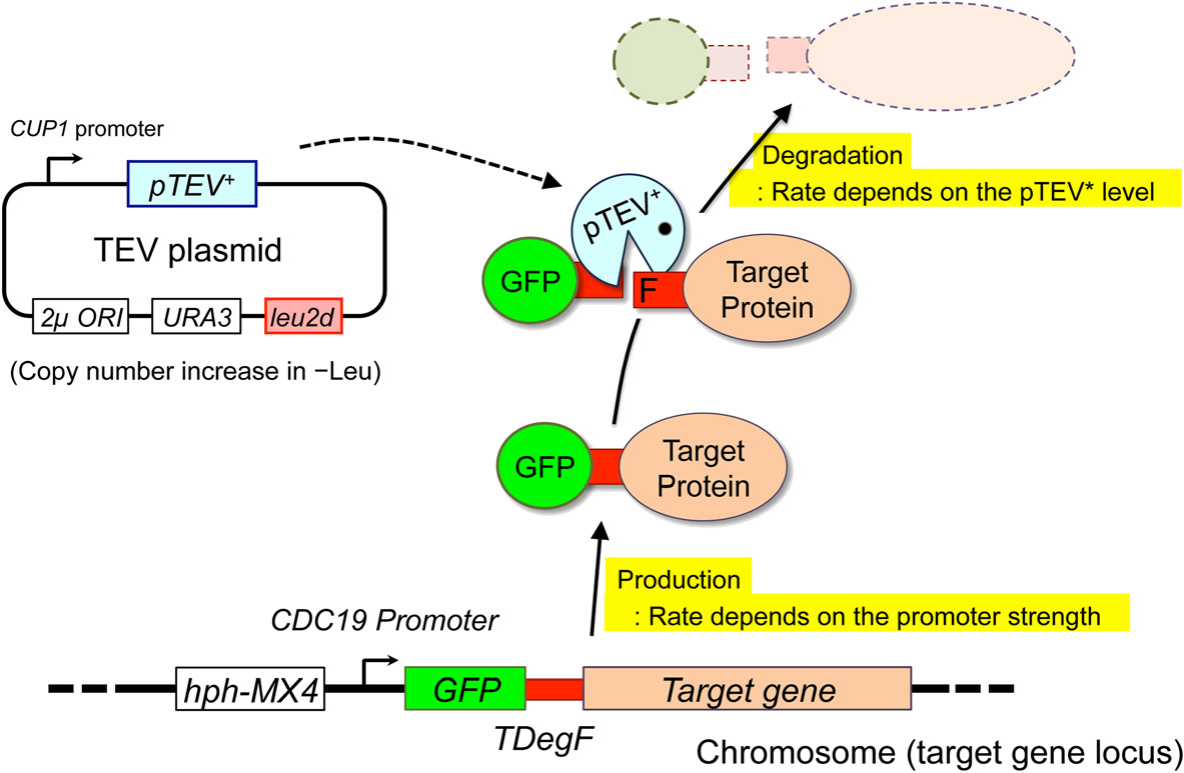
**Scheme of TIPI-gTOW.** We first constructed a strain in which the chromosomal target gene was replaced by a *GFP-*^*TDegF*^*target* construct. We next introduced the TEV plasmid, a plasmid for gTOW that encodes pTEV^+^ expressed from the *CUP1* promoter. According to the TIPI procedure, cleavage and rapid degradation of the GFP-^TDegF^target is induced by pTEV^+^. Using the gTOW procedure, in which the copy number of the TEV plasmid exceeds 100 under the -Leu condition, we can increase the expression of pTEV^+^, which accelerates the degradation of the GFP-^TDegF^target, reducing the level of the GFP-^TDegF^target. It is thus expected that the upper limit copy number of the TEV plasmid would inversely correlate with the lower limit of the GFP-^TDegF^target. The tug-of-war between the bias to increase the copy number of *leu2d* and the bias to decrease the copy number of pTEV^+^ gene determines the plasmid copy number in the cell under the -Leu condition. It is thus possible to indirectly estimate the lower limit of the GFP-^TDegF^target by measuring the copy number of the TEV plasmid.

In the gTOW method, we cloned the target gene into a plasmid and increased the copy number using the genetic bias for *leu2d*[7,8]. In this study, by increasing the copy number of the gene encoding TEV protease using the gTOW scheme, we attempted to increase the degradation rate of a target protein containing a TEV protease-induced degron (Figure 1). If the target protein expression is at the lower limit for any essential cellular function, the degradation rate must be less than that required to maintain the lower limit, which will restrict the upper limit copy number of the gene encoding TEV. We designated this scheme TIPI-gTOW.

To ensure the success of this approach, we need to adjust the expression of the following: (1) the target protein (determined by the production and degradation rate) to be in the range required to support the growth of the cell (not too high and not too low) and (2) the TEV protease to be in the appropriate range to induce degradation in order to detect the lower limit of the target protein expression. Taxis *et al.* used the *CYC1* promoter and *ADH1* promoter to express target proteins and the *GAL1* promoter to induce TEV protease expression [22,23]. In some cases, they could not observe expected lethal phenotypes, probably because the induced degradation of the target proteins by the TEV protease was insufficient to reduce the protein expression to their lower limits [22,23].

We used constitutive promoters to increase the expression of TEV protease in accordance with the increase in gene copy number. We first used the *S. cerevisiae TEF1* promoter to express TEV protease, but the strong expression of TEV protease from the *TEF1* promoter on the high-copy gTOW plasmid in itself caused a cellular growth defect (data not shown). We currently do no know what causes this toxicity. We then used the *CUP1* promoter. In the culture conditions we used (0.25 μM Cu^2+^), the *CUP1* mRNA level was about 10% of the *TEF1* mRNA level (data not shown). Expression of the TEV protease from the *CUP1* promoter did not show any cellular growth defect, even when the gene copy number exceeded 100 (data not shown). We used an efficient version of the p14–TEV fusion protein (p14*–TEV^+^) [22,23]; here, we designated this “pTEV^+^.” We also designated the gTOW plasmid encoding the pTEV^+^ expressed from the *CUP1* promoter as the “TEV plasmid.”

**Figure 2:**
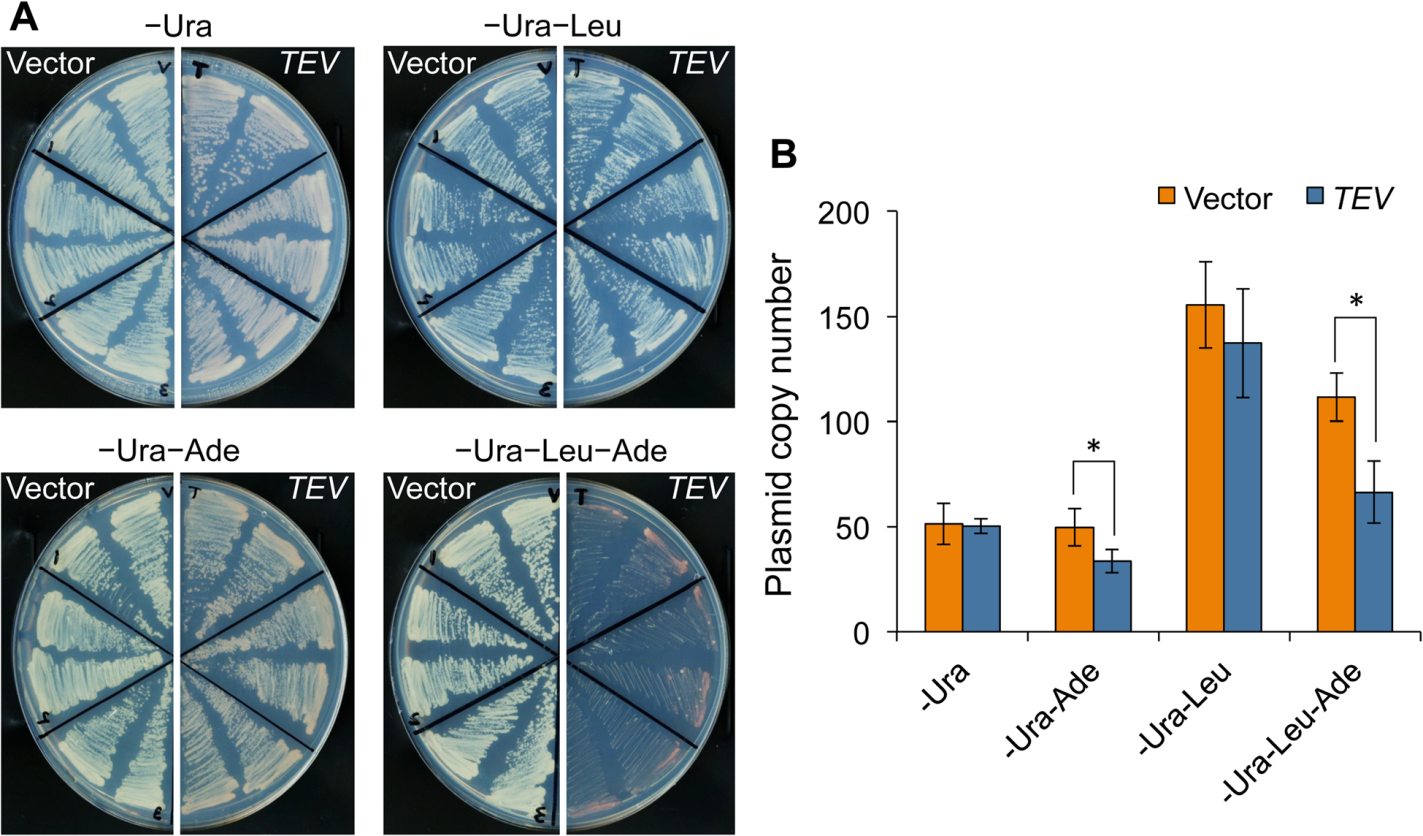
**Evaluation of the lower limit of Ade2 using TIPI-gTOW. (A)** Growth of the cells harboring *GFP-*^*TDegF*^*ADE2* (YSM001) with the vector and the TEV plasmid on SC plates without the indicated amino acids. Six independent colonies of each strain were tested. **(B)** Copy numbers of the plasmids of *GFP-*^*TDegF*^*ADE2* (YSM001) in SC medium without the indicated amino acids. Four independent measurements were performed, and the average is shown. The error bar indicates the standard deviation. *: *p* < 0.01 for Student’s *t*-test.

To test the feasibility of TIPI-gTOW, we selected *ADE2* as a target. *ADE2* encode phosphoribosylaminoimidazole carboxylase, an enzyme involved in the adenine synthesis. Reduction of Ade2 protein can be indirectly monitored by assessing cellular growth defect and by accumulation of red pigment in the absence of adenine [25]. We integrated *ADE2* with the TEV-induced degron expressed from the *CDC19* promoter into the chromosomal *ADE2* locus (Figure 1). We chose the *TDegF* degron because it is one of the strongest degrons [22,23]. We then introduced the TEV plasmid into the cell. The results of TIPI-gTOW experiments of *ADE2* are shown in Figure 2. Cellular growth deficiency and red colony formation were observed for cells containing the TEV plasmid in the -Leu-Ade condition (Figure 2A). We note that colonies are expected to be redder on SC–Ura–Leu plates than the ones on SC–Ura plates, but they were not (Figure 2A). We currently do not know the reason. Unknown interaction between the leucine deficiency and the red color formation might exist. We next measured the copy numbers of the TEV plasmid and found that the copy number limit of the TEV plasmid decreased significantly under the -Leu-Ade condition (Figure 2B). We thus ensured that the Ade2 protein was expressed at the lower limit and indirectly estimated the lower limit from the TEV plasmid copy number. We note that the copy numbers of the vector were significantly different in between –Ura–Leu and –Ura–Leu–Ade conditions (p < 0.01, Student’s *t-*test). The replication and/or partition of the 2 μ-based plasmid might be affected by the growth conditions.

### TIPI-gTOW experiments of cell cycle regulators

We next selected 3 essential cell cycle regulators as target genes to test if TIPI-gTOW was a feasible approach to assess their lower limits. We constructed the *GFP-TDegF* constructs for *CDC15*, *CDC20*, and *CDC28* and integrated these into their chromosomal loci. At first, we used the full-length *CDC19* promoter (729 bp) to express the cell cycle proteins but could not obtain a *CDC20* construct, probably because strong expression of *CDC20* is toxic [26]. We then constructed a series of deletions of the *CDC19* promoter to reduce its expression (Additional file 1: Figure S1). The GFP expression from promoters with a length of 500 bp or less was markedly lower than that from promoters exceeding 500 bp (Additional file 1: Figures S1B and S1C). This reduction might be due to the deletion of a Tye7 binding site located at -523 bp to -516 bp (Additional file 1: Figure S1A). We thus used the full-length *CDC19* promoter (729 bp), the *CDC19*_*-600*_ promoter (600 bp), and the *CDC19*_*-500*_ promoter (500 bp) to construct the *GFP-TDegF* target.

**Figure 3:**
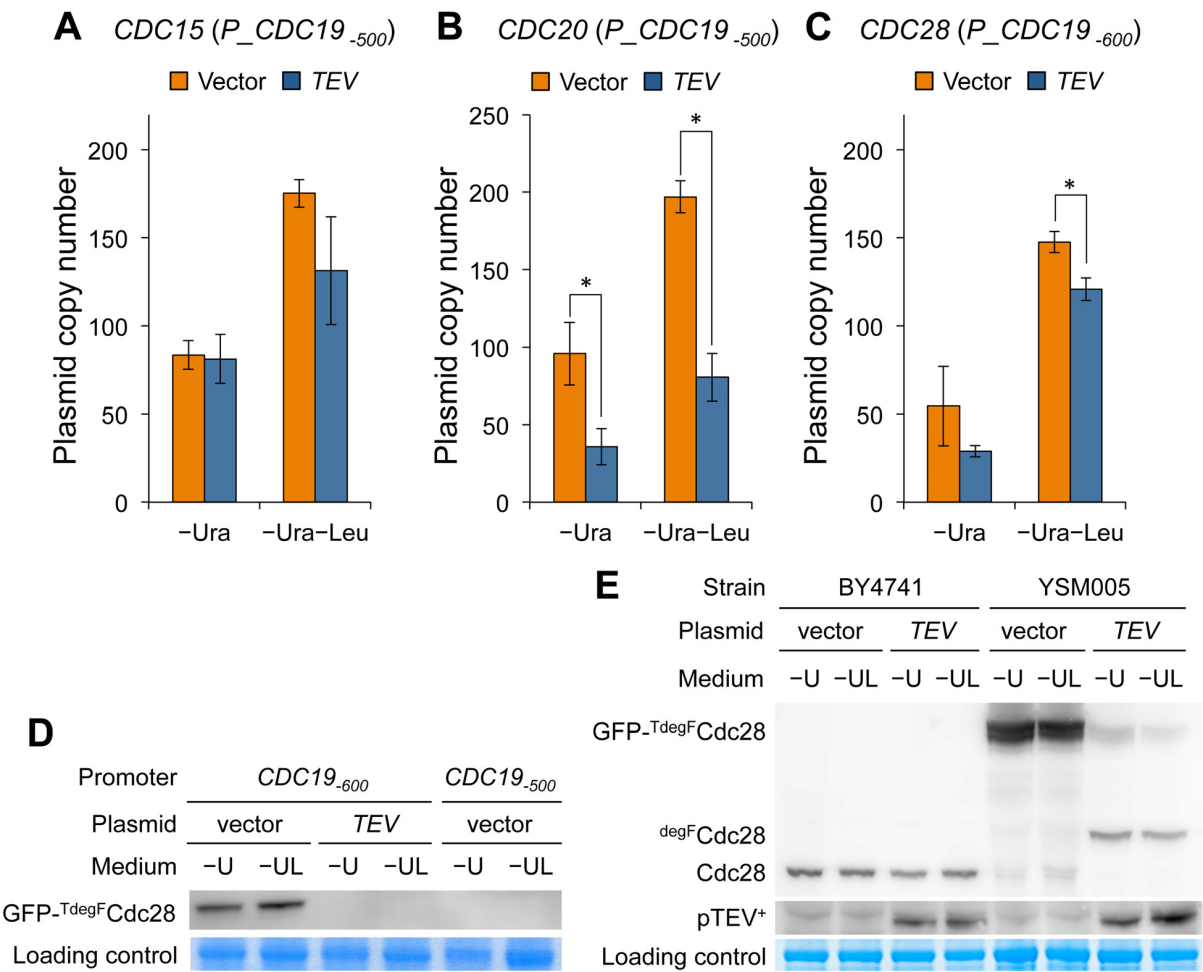
**TIPI-gTOW experiments of the cell cycle regulators.** Copy numbers of the plasmids in the strain; **(A)***P_CDC19*_*-500*_*-GFP-*^*TdegF*^*CDC15* (YSM002), **(B)***P*_*CDC19*_*-500*_-*GFP-*^*TdegF*^*CDC20* (YSM003), and **(C)***P_CDC19*_*-600*_*-GFP-*^*TdegF*^*CDC28* (YSM005) in SC medium without the indicated amino acids. Four independent measurements were performed, and the average is shown. The error bar indicates the standard deviation. *: *p* < 0.01 for Student’s *t*-test. **(D)** Western blotting for GFP-^TdegF^Cdc28 protein in strains YSM005 (*P_CDC19*_*-600*_) and YSM004 (*P_CDC19*_*-500*_) expressed in SC medium without the indicated amino acids, as detected with anti-GFP antibody. **(E)** Western blotting for GFP-^TdegF^Cdc28 protein and pTEV^+^ in strains BY4741 (wild type) and YSM005 (*P_CDC19*_*-600*_*-GFP-*^*TdegF*^*CDC28*) expressed in SC medium without the indicated amino acids, as detected with anti-Cdc28 antibody (upper blot) and anti-TEV protease antibody (middle blot). In **D** and **E**; Coomassie® G-250 staining of 50 kDa bands (corresponding to the size of EF-1α) are shown as loading controls. “–U” and “–UL” indicate –Ura and –Ura–Leu, respectively.

The results of the constructions are summarized in Additional file 1: Table S1. GFP-^*TDegF*^*CDC15* constructs with all 3 promoters were obtained. We observed reduction in the copy number of the TEV plasmid with the *CDC19*_*-500*_ promoter construct, but this was not significant (Figure 3A, *p* > 0.01, Student’s *t*-test). The lower limit of Cdc15 may be lower than that produced by TIPI-gTOW. As described above, we could not obtain a *GFP-*^*TDegF*^*CDC20* construct with the full-length *CDC19* promoter or the *CDC19*_*-600*_ promoter probably because the expression was too high. However, we obtained a *GFP-*^*TDegF*^*CDC20* integrated strain with the *CDC19*_*-500*_ promoter. With this strain, we succeeded to detect significant restriction of the copy numbers of the TEV plasmid under both -Ura and -Ura-Leu conditions (Figure 3B, *p* < 0.01, Student’s *t*-test). We obtained GFP-^*TDegF*^*CDC28* integrated strains with all the 3 promoters and observed significant restrictions of the copy number of the TEV plasmid with the *CDC19*_*-600*_ promoter under the - Ura - Leu condition (Figure 3C, *p* < 0.01, Student’s *t*-test). We did obtain a GFP-^*TDegF*^*CDC28* integrated strain with the *CDC19*_*-500*_ promoter but could not obtain any transformants with the TEV plasmid, probably because the expression of Cdc28 from the *CDC19*_*-500*_ promoter was already close to the lower limit of Cdc28, even in the absence of TEV protease.

Reduction in the protein expression of GFP-^TDegF^Cdc28 by TEV protease was confirmed by western blotting with anti-GFP antibody (Figure 3D). The GFP-^TDegF^Cdc28 protein expressed from the *CDC19*_*-500*_ promoter seemed to be too low to be detected by western blotting (Figure 3D). Simultaneously, we could not detect any GFP-^TDegF^Cdc20 protein expressed from the *CDC19-500* promoter (data not shown).

We further analyzed the TEV protease dependent cleavage and reduction of GFP-^TDegF^Cdc28 by western blotting with anti-Cdc28 antibody (Figure 3E). The protein levels of GFP-^TDegF^Cdc28 in YSM005 (*P_CDC19*_*-600*_*-GFP-*^*TDegF*^*CDC28*) were highly reduced in the presence of the TEV plasmid compared with the vector control, and the level was lower under the –Ura–Leu condition, where the expression of pTEV^+^ increased, than the –Ura condition. Cleaved products (^degF^Cdc28) were also observed in the presence of the TEV plasmid. The summed levels of the GFP-^TDegF^Cdc28 and ^degF^Cdc28 were comparable with the Cdc28 levels expressed in BY4741 (wild type). These results confirmed that the target protein level was actually reduced using TIPI-gTOW. We also tired to detect Cdc20 by western blotting using anti-Cdc20 antibodies (sc-6730 and sc-6731, Santa Cruz biotechnology). However, we could not detect any signal, probably because the expression level of Cdc20 is too low to detect with these antibodies.

### Estimation of changes in the lower limit of *CDC20* in gene-deletion strains

As described above, we identified conditions under which the GFP-^TDegF^Cdc20 expression was reduced to the lower limit. We next tried to estimate the lower limits of GFP-^TDeg^Cdc20 in gene-deletion strains using TIPI-gTOW. We integrated the *P_CDC19*_*-500*_*-GFP-*^*TDegF*^*Cdc20* construct into the chromosomal *CDC20* locus of each of 23 cell cycle regulator deletion strains and measured the TEV plasmid copy number. The copy number data is shown in Table 1. We first noticed that the copy numbers of the vector itself varied among deletion strains. This may be because of differences in the growth rate or the efficiency of replication and partitioning of the plasmid among the deletion strains.

**Table 1:** **Copy numbers of the vector and the TEV plasmid in *CDC20 *** **TIPI-gTOW in the gene-deletion strains**

	Vector copy number*		TEV plasmid copy number*		**TEV/vector ratio**	**Relative change in copy number**
	**Average**	**SD**	**Average**	**SD**		
Wild type	149.7	43.4	59.8	19.9	0.40	–
*bck2Δ*	44.3	3.4	25.3	0.8	0.57	0.43
*bub2Δ*	71.4	8.4	48.6	3.5	0.68	0.70
*cdh1Δ*	66.7	5.7	19.6	2.2	0.29	-0.27
*cin8Δ*	53.7	20.0	37.1	5.0	0.69	0.73
*clb1Δ*	57.6	5.2	19.2	2.2	0.33	-0.16
*clb2Δ*	56.3	4.3	20.2	7.8	0.36	-0.10
*clb3Δ*	77.5	15.1	27.7	3.6	0.36	-0.11
*clb4Δ*	59.4	4.5	19.3	2.1	0.32	-0.19
*clb5Δ*	98.0	21.4	33.6	6.5	0.34	-0.14
*clb6Δ*	76.1	8.6	31.6	2.7	0.42	0.04
*cln1Δ*	80.1	24.3	20.7	0.6	0.26	-0.35
*cln2Δ*	82.7	15.8	42.9	3.8	0.52	0.30
*cln3Δ*	47.0	10.9	22.0	10.8	0.47	0.17
*lte1Δ*	84.8	20.9	32.0	16.0	0.38	-0.06
*mad2Δ*	47.4	6.0	35.2	2.9	0.74	0.86
*mbp1Δ*	21.6	11.4	15.0	2.1	0.69	0.73
*mih1Δ*	64.4	12.1	29.3	3.7	0.46	0.14
*sic1Δ*	71.8	20.1	20.2	11.8	0.28	-0.29
*swe1Δ*	124.4	13.0	42.2	5.3	0.34	-0.15
*swi4Δ*	76.1	15.1	28.4	6.6	0.37	-0.07
*swi5Δ*	94.0	23.8	37.3	12.6	0.40	-0.01
*swi6Δ*	64.0	8.7	35.2	7.2	0.55	0.38
*whi5Δ*	69.6	15.8	33.8	10.9	0.49	0.22

*Average and SD of four independent experimental trials are shown.

In addition, we observed significant correlations in the copy numbers of the vector and the TEV plasmid under both -Ura (*r* = 0.61) and -Ura-Leu (*r* = 7.0) conditions (Additional file 1: Figure S2). We thus considered that it was not appropriate to simply estimate the lower limit of GFP-^TDegF^Cdc20 from the copy number of the TEV plasmid in each deletion strain. Therefore, we calculated the ratio of the copy number of the TEV plasmid to that of the vector in each deletion strain and compared the ratio with that of the wild type to estimate the relative change in TEV plasmid copy number (see Methods for details).

**Figure 4:**
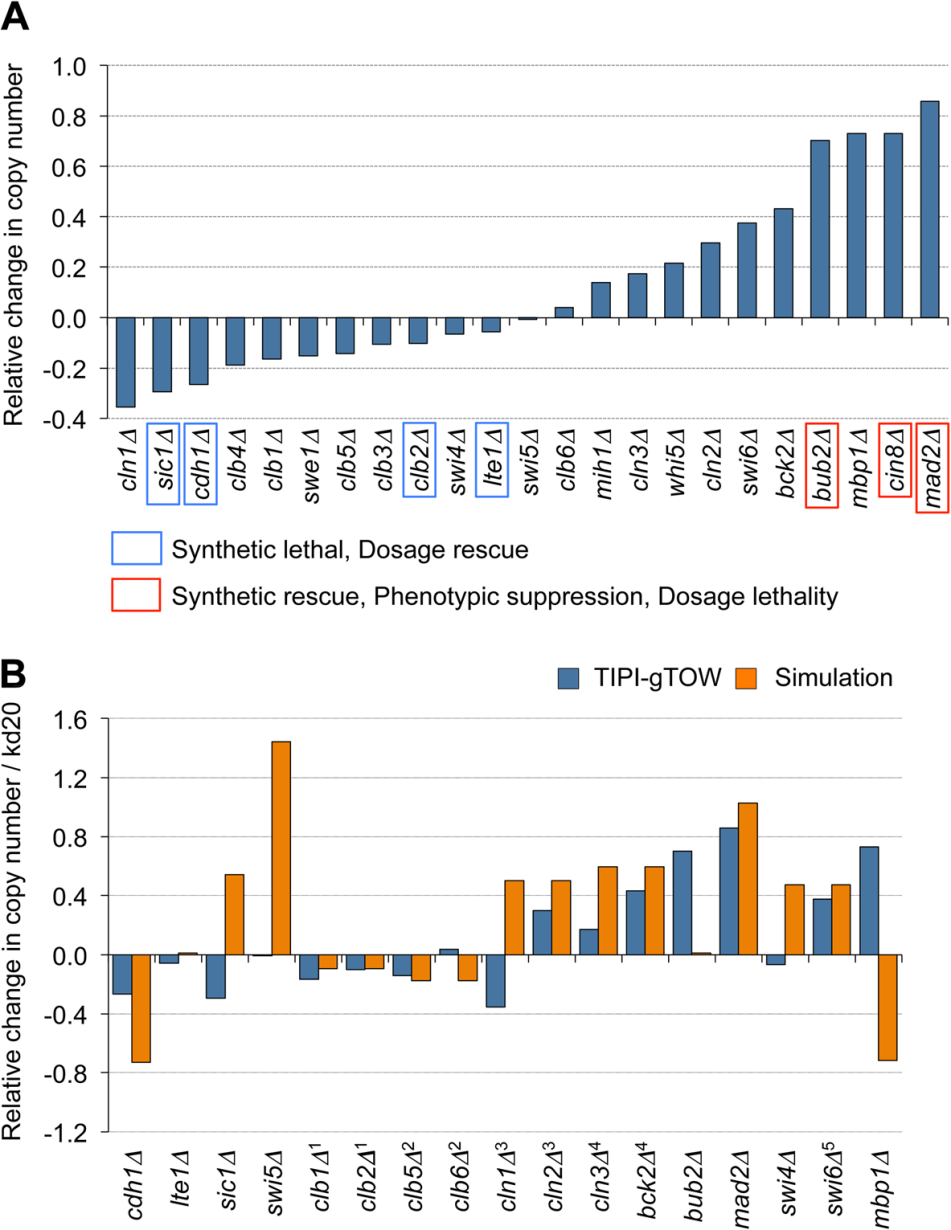
**Evaluation of the lower limits of Cdc20 protein on deletion of cell cycle regulators. (A)** Relative copy number changes in the TEV plasmids in gene-deletion strains with *GFP-*^*TdegF*^*CDC20*. Genes with previously identified genetic interactions with *cdc20* mutation were boxed (summarized in Table 2). The original data used for the graph is shown in Table 1. **(B)** Relative copy number changes in TEV plasmids in gene-deletion strains with *GFP-*^*TdegF*^*CDC20* selected from **A** and simulation results. The degradation rate of kd20 is gradually increased until the cell cycle simulation indicates “dead.” In the simulation, 1: Clb1 and Clb2, 2: Clb5 and Clb6, 3: Cln1 and Cln2, and 4: Cln3 and Bck2 are implemented as single genes. 5: Swi6 is implemented into the component of SBF (together with Swi4) and MBF (together with Mbp1). The original data used for the graph is shown in Table 1 and Additional file 1: Table S2.

Figure 4A and Table 1 show the relative copy number change in each deletion strain. Some strains, such as *cln1*∆, *sic1*∆, and *cdh1*∆, had negative relative copy number change values, which indicated that the copy numbers of the TEV plasmids in these strains were lower than those in the wild type, indicating that the lower limits of GFP-^TDegF^Cdc20 increased in these strains. This implies that the deleted genes are involved in the production or activation of Cdc20 or have functions overlapping those of Cdc20 (Additional file 1: Figure S3). In these cases, the genes could be synthetically lethal with *cdc20* mutants or multicopy suppressors of *cdc20* mutants.

Other strains, such as *mad2*∆, *cin8*∆, and *mbp1*∆, showed positive relative copy number change values, indicating that the copy numbers of the TEV plasmids were higher than those in the wild type; thus, the lower limits of GFP-^TDegF^Cdc20 decreased in these strains. These deleted genes are therefore involved in inhibition of Cdc20, e.g., by inhibition of Cdc20 production or Cdc20 activity or degradation of Cdc20 (Additional file 1: Figure S4). These genes could be dosage suppressors of the toxicity of Cdc20 overexpression.

Previous studies, including systematic analyses, identified genes showing genetic interactions with *CDC20*[13,27-30]; these were listed in the *Saccharomyces* Genome Database (yeastgenome.org). From these interactions, we summarized relevant genetic interactions studied here in Table 2 and Figure 4A. Importantly, the relative copy number of the TEV plasmid decreased in deletion strains involving genes showing synthetic lethality or dosage rescue of the *cdc20* mutant, whereas the relative copy number of the TEV plasmid increased in deletion strains involving genes showing synthetic rescue, phenotypic suppression, and dosage lethality of the *cdc20* mutant. This result indicated that our TIPI-gTOW approach gave information regarding genetic interactions, which was highly consistent with the previously identified interactions. Moreover, the advantage of our approach is that we could isolate genetic interactions in both directions, namely negative interactions such as synthetic lethal and dosage rescue and positive interactions such as synthetic rescue, phenotypic suppression, and dosage lethality in a single experiment.

**Table 2:** **Known genetic interactions with *CDC20 *** **mutation listed in the *Saccharomyces *** Genome Database*

**Gene**	**Genetic interaction with the *cdc20 *** **Mutant**	**Description**	**References**
*BUB2*	Synthetic Rescue	Temperature sensitivity of *cdc20-1*(ts) is rescued by *bub2* deletion.	[27]
*CDH1*	Synthetic Lethality	*cdc20-1*(ts) shows synthetic lethality with *cdh1* deletion.	[28]
*CIN8*	Dosage Lethality	In the absence of *CIN8*, overexpression of *CDC20* is lethal.	[29]
*CLB2*	Negative Genetic	Growth defect of *cdc20_tsq368* increases with *clb2* deletion.	[13]
*LTE1*	Synthetic Lethality	*cdc20-1*(ts) shows synthetic lethality with *lte1* deletion.	[28]
*MAD2*	Phenotypic Suppression	*cdc20* blocks the premature degradation of Pds1 observed in a *mad2* mutant.	[30]
*SIC1*	Synthetic Lethality	*cdc20-1*(ts) shows synthetic lethality with *sic1* deletion.	[28]

*When synonymous genetic interactions were reported in more than one study, one representative is listed.

Among genetic interactions obtained in this study, the difference between *CLN1* and *CLN2* was surprising because they are known to have heavily overlapped functions [31]. Previous analysis, however, showed that *CLB4* has opposite genetic interactions with *CLN1* and *CLN2*[32]. Our results might indicate that these genes have different functions in some contexts.

Comparison of experimental data with predictions of a simulation model is useful for evaluating the predictive ability of the mathematical model and for speculating about molecular mechanisms generating negative/positive genetic interactions [7,9,10]. An integrative mathematical model of the budding yeast cell cycle was developed previously [33], and we have modified this model using gTOW data [9]. Using the “stabilization model” published in the reference [9], we here measured how much the degradation parameter of Cdc20 could be increased (i.e., the upper limit of kd20) in each of the gene-deletion models and compared the upper limit of kd20 with that in the wild type to obtain the relative change in the upper limit of kd20 (see Methods for details). This analysis is considered to represent the process of TIPI-gTOW, namely indirectly assessing the lower limits of Cdc20 in the presence of gene deletions, by measuring how much degradation can increase without halting the cell cycle. The simulation result is shown in Additional file 1: Table S2. Figure 4B shows the comparison of the results of TIPI-gTOW and those of the simulation. Deletion strains, such as *mad2Δ*, *cdh1Δ*, and others, showed good agreements between TIPI-gTOW and simulation data; however, some genes such as *sic1Δ*, *swi5Δ*, and *mbp1Δ* showed almost opposite changes, suggesting absence of some regulatory steps in the model.

## Conclusions

In this study, we tried to develop a method for estimating the lower limit of protein expression by combining the TIPI and gTOW methods. Using TIPI-gTOW, we successfully constructed a strain in which GFP-^TDegF^Ade2 was expressed at the lower limit, just sufficient to support cellular growth under the -Ade condition by accelerating degradation by TEV protease.

We also succeeded in constructing a strain in which the minimal level of GFP-^TDegF^Cdc20 was expressed by TIPI-gTOW. Using this strain, we studied genetic interactions between cell cycle regulators and *CDC20*. We concluded that TIPI-gTOW is useful for estimating changes in the lower limit of a protein under different conditions, such as different genetic backgrounds and environments. TIPI-gTOW is useful for analyzing genetic interactions of essential genes whose deletion mutants cannot be obtained. One important characteristic of TIPI-gTOW is that it is useful for identifying both negative and positive genetic interactions, as shown in Figure 4. The synthetic genetic array is a potent high-throughput approach to identifying genetic interactions [34], which now allows quantitative analyses [35] and can also applied to essential genes [36]. TIPI-gTOW developed in this study may be an alternative method to study genetic interactions in detail.

The conventional gTOW method developed previously uses a gene with a native promoter and terminator as a unit to increase protein expression and measures the copy number limit to estimate the “copy number limit for protein overexpression” [7,8,10,11]. The advantage of gTOW is that we can indirectly estimate by how much a target protein can be overexpressed over the native level by simply measuring the copy number of the gTOW plasmid. In TIPI-gTOW, however, we first have to modify the native copy of the target gene, which changes the native expression, activity, and stability of the target protein. In addition, the aim of the procedure is to reduce the expression of the target protein, which makes it difficult to quantitate the lower limit of the target protein by western blotting when this level is under the detection limit. In fact, we have failed to detect the protein levels of Ade2 and Cdc20 by western blotting using their specific antibodies (data not shown). We thus believe that TIPI-gTOW is a method by which a cellular condition can be constructed with the target protein expressed at the lower limit still supporting cellular function. Thus, it is useful for identifying genetic interactions as described above, although it does not measure the absolute lower limit of the target protein.

To use TIPI-gTOW, the optimal expression for the target GFP-TDegF protein must be determined so that the level is within the range of detection of the lower limit by increasing the TEV plasmid. Taxis and Knop developed a series of TDeg constructs with different promoters and N-degrons of various strengths [23], which will be useful to systematically determine the optimal expression.

Previous studies on haploinsufficiency and dosage compensation suggested that most of yeast proteins are expressed at least twice more than the levels required in diploid cells [37,38]. If we could detect the lower limit protein levels under the TIPI-gTOW experiments, we might be able to argue about this issue. We failed to detect some proteins using western blotting with specific antibodies as described above. Fusing the target proteins with more sensitive proves might be useful.

## Methods

### Yeast strains and growth conditions

Yeast strains used in this study are listed in Table 3. A *S. cerevisiae* strain BY4741 (*MATa*, *his3Δ1*, *leu2Δ0*, *met15Δ0*, *ura3Δ0*) [39] was used as the host strain for the experiments. BY4741 derivatives with gene deletions in the cell cycle regulators were obtained from Open Biosystems/Thermo Scientific. YSM001 was constructed by integrating the *P_CDC19*-*GFP-*^*TDegF*^*ADE2* fragment, which was amplified by PCR with the primers OHML432 and OHML389 using pSS1002 as a template, into the chromosomal *ADE2* locus of BY4741. Similarly, YSM002 was constructed by integrating the *P_CDC19*_*-500*_-*GFP-*^*TDegF*^ fragment, which was amplified by PCR with the primers OHML504 and OHML505 using pSM001 as a template, into the chromosomal *CDC15* locus of BY4741. YSM003 was constructed by integrating the *P_CDC19*_*-500*_-*GFP-*^*TDegF*^ fragment, which was amplified by PCR with the primers OHML506 and OHML507 using pSM001 as a template, into the chromosomal *CDC20* locus of BY4741. YSM004 was constructed by integrating the *P_CDC19*_*-500*_-*GFP-*^*TDegF*^ fragment, amplified by PCR using the primers OHML508 and OHML509 and pSM001 as a template, into the chromosomal *CDC28* locus of BY4741. YSM005 was constructed as for YSM004, except that pSM002 was used as a PCR template. Each *P_CDC19*_*-500*_-*GFP-*^*TDegF*^*CDC20* integrated strain harboring a gene deletion was constructed by integrating the same DNA fragment as YSM003 into the chromosomal *CDC20* locus of each deletion strain. The sequences of PCR primers are listed in Additional file 1: Table S3. Yeast cells were cultured as described previously [9,40]. Yeast cells were cultured in SC media without uracil (Ura), adenine (Ade), leucine (Leu), and histidine (His) as indicated. 2% glucose was used as a carbon source. SC media were made using YNB with ammonium sulfate (MP biomedicals) that contains 0.25 μM Cu^2+^.

**Table 3:** Yeast strains used in this study

**Name**	**Genotype**	**Source**
BY4741	*MAT*a, *his3Δ1*, *leu2Δ0*, *met15Δ0*, *ura3Δ0*	[39]
YSM001	*ade2::hphMX-P_CDC19-GFP-*^*TDegF*^*ADE2* in BY4741	This study
YSM002	*cdc15::hphMX-P_CDC19*_*-500*_*-GFP-*^*TDegF*^*CDC15* in BY4741	This study
YSM003	*cdc20::hphMX-P_CDC19*_*-500*_*-GFP-*^*TDegF*^*CDC20* in BY4741	This study
YSM004	*cdc28::hphMX-P_CDC19*_*-500*_*-GFP-*^*TDegF*^*CDC28* in BY4741	This study
YSM005	*cdc28::hphMX-P_CDC19*_*-600*_*-GFP-*^*TDegF*^*CDC28* in BY4741	This study

### Plasmids used in this study

Plasmids used in this study are listed in Table 4. pSS1006 (the TEV plasmid) was constructed by inserting p14^*^–TEV^+^ from pDS5 [22] into pSBI40 [7] so that p14^*^–TEV^+^ is expressed under control of the *CUP1* promoter. pSS1002 was constructed by connecting *hph-MX4*, a hygromycin B-resistance cassette [41], the *CDC19* promoter, *GFP*-*TDegF* from pDS41 [22], and *ADE2*, followed by insertion into pRS423ks [11]. pSS1002, with different lengths of *CDC19* promoters, was constructed using the PCR primers OHML541–547 and OHML540; the sequences are listed in Additional file 1: Table S3.

**Table 4:** Plasmids used in this study

**Name**	**Relevant characteristics**	**Source**
pSBI40	*2 μ ori*, *URA3*, *leu2d*; plasmid for gTOW experiment	[7]
pRS423ks	*2 μ ori*, *HIS3*	[11]
pDS5	p14*–TEV^*+*^	[22]
pDS41	*GFP-TDegF-SF3b*	[22]
pSS1002	*hph-MX4*, *P_CDC19-GFP-*^*TDegF*^*ADE2* in pRS423ks	This study
pSS1006	P_CUP1-P14*–TEV^*+*^ in pSBI40	This study
pSM001	*P_CDC19*_*-500*_ instead of *P_CDC19* in pSS1002	This study
pSM002	*P_CDC19*_*-600*_ instead of *P_CDC19* in pSS1002	This study

### Copy number determination of plasmids

The copy number of a plasmid in a cell was measured as described previously [7] with some modifications. Cells of each yeast strain with pSS1006 (the TEV plasmid) or pSBI40 (the empty vector plasmid) were grown for 50 h in 200 μL SC medium without relevant amino acids. The cells were corrected and suspended into 50 μL of Zymolyase solution (10 mM Na-phosphate [pH 7.5], 1.2 M sorbitol, and 2.5 mg/ml Zymolyase 100 T (Nacalai tesque)). The cell suspension was incubated for 15 min at 37°C, and then incubated for 10 min at 100°C. After removing the cell debris by centrifugation, the supernatant was used as the total DNA solution. Two real-time PCRs were performed using 2 μL of the DNA solution as templates with *LEU2* and *LEU3* primer sets (primer sequences are listed in Additional file 1: Table S3), using LightCycler® 480 SYBR Green I Master with the LightCycler® 480 system (Roche). The *LEU2* and *LEU3* primer sets were used to quantify the *LEU2* DNA from the plasmid and the *LEU3* DNA from the genome, respectively. The plasmid copy number per haploid genome was estimated by comparing the relative amount of the *LEU2* DNA and the *LEU3* DNA as follows: $\mathrm{Plasmid}\;\mathrm{copy}\;\mathrm{number}=2^{{}^{\left(\mathrm{Cp}\_\mathrm{LEU}2-\mathrm{Cp}\_\mathrm{LEU}3\right)}}$, where Cp_LEU2 and Cp_LEU3 are the crossing points of PCR amplifications using the *LEU2* and *LEU3* primer sets, respectively.

The relative copy number change of the TEV plasmid was calculated as follows: Relative copy number change = (*ΔC*_*T*_/*C*_*V*_ - *wtC*_*T*_/*wtC*_*V*_)/(*wtC*_*T*_/*wtC*_*V*_), where *ΔC*_*T*_ is the average copy number of the TEV plasmid in each knockout strain, *ΔC*_*V*_ is the average copy number of the empty vector plasmid in each knockout strain, *wtC*_*T*_ is the average copy number of the TEV plasmid in the wild-type strain, and *wtC*_*V*_ is the average copy number of the empty vector plasmid in the wild-type strain.

### Western blotting

Cells were cultivated in SC medium, and the proteins were extracted as described previously [7]. The proteins were separated using NuPAGE® 4-12% Bis-Tris gels, and transferred them onto a PVDF membrane using iBlot® gel transfer system (Life Technologies), then the target proteins were detected using their specific antibodies. For the detection of GFP, TEV protease, and Cdc28, antibodies 1814460 (Roche), PAB19931 (Abnova), and sc-6709 (Santa Cruz Biotechnology) were used, respectively. For the second antibodies, Histofine® simple stain MAX PO (MULTI) and Histofine® simple stain MAX PO (G)(Nichirei) were used. Coomassie® G-250 (SimplyBlue™ SafeStain, Life Technologies) was used to stain loading controls.

### Computation

Numerical simulations were performed using Matlab version 7.3.0 with the “stabilization model” described previously [9]. The rate of degradation of kd20 was gradually increased until the cell cycle simulation yielded the result “dead.” The relative degradation rate change was calculated as follows: Relative degradation rate change = (*ΔUL*_*kd20*_ - *wtUL*_*kd20*_)/*wtUL*_*kd20*_, where *ΔUL*_*kd20*_ is the upper limit of kd20 in a deletion mutant model and *wtUL*_*kd20*_ is that in the wild-type model.

## Competing interests

The authors declare that they have no competing interests.

## Authors’ contributions

MS mainly carried out the molecular genetic experiments in this study. SS carried out the initial construction of TIPI-gTOW. KK carried out computational analysis. KM participated in the constructions of plasmids. RK performed western blotting. HM conceived of the study, and participated in its design and coordination and draft the manuscript. All authors read and approved the final manuscript.

## Supplementary Material

Additional file 1 This file contains 3 tables (**Table S1.** Summary of construction of *GFP-DegF* integrated strains with different lengths of *CDC19* promoters. **Table S2.** Upper limits of the rates of degradation of Cdc20 (kd20) in gene-deletion models. **Table S3.** PCR primers used in this study.) and 4 figures **(Figure S1.** Serial deletions of the *CDC19* promoter. **Figure S2.** Correlation between the copy numbers of vector and TEV plasmids. **Figure S3.** Potential outcomes of TIPI-gTOW of Cdc20 in cell cycle regulator deletion strains and their regulatory interactions (Case 1). **Figure S4.** Potential outcomes of TIPI-gTOW of Cdc20 in cell cycle regulator deletion strains and their regulatory interactions (Case 2)). Click here for file

## References

[B1] Zaslaver A Mayo AE Rosenberg R Bashkin P Sberro H Tsalyuk M Surette MG Alon U Just-in-time transcription program in metabolic pathways Nat Genet 2004 36 486 491 10.1038/ng1348 15107854

[B2] Dekel E Alon U Optimality and evolutionary tuning of the expression level of a protein Nature 2005 436 588 592 10.1038/nature03842 16049495

[B3] Wagner A Energy constraints on the evolution of gene expression Mol Biol Evol 2005 22 1365 1374 10.1093/molbev/msi126 15758206

[B4] Alon U Surette MG Barkai N Leibler S Robustness in bacterial chemotaxis Nature 1999 397 168 171 10.1038/16483 9923680

[B5] Little JW Shepley DP Wert DW Robustness of a gene regulatory circuit EMBO J 1999 18 4299 4307 10.1093/emboj/18.15.4299 10428968 PMC1171506

[B6] von Dassow G Meir E Munro EM Odell GM The segment polarity network is a robust developmental module Nature 2000 406 188 192 10.1038/35018085 10910359

[B7] Moriya H Shimizu-Yoshida Y Kitano H In vivo robustness analysis of cell division cycle genes in Saccharomyces cerevisiae PLoS Genet 2006 2 e111 10.1371/journal.pgen.0020111 16839182 PMC1500812

[B8] Moriya H Makanae K Watanabe K Chino A Shimizu-Yoshida Y Robustness analysis of cellular systems using the genetic tug-of-war method Mol Biosyst 2012 8 2513 2522 10.1039/c2mb25100k 22722869

[B9] Kaizu K Moriya H Kitano H Fragilities caused by dosage imbalance in regulation of the budding yeast cell cycle PLoS Genet 2010 6 e1000919 10.1371/journal.pgen.1000919 20421994 PMC2858678

[B10] Moriya H Chino A Kapuy O Csikász-Nagy A Novák B Overexpression limits of fission yeast cell-cycle regulators in vivo and in silico Mol Syst Biol 2011 7 556 22146300 10.1038/msb.2011.91 PMC3737731

[B11] Makanae K Kintaka R Makino T Kitano H Moriya H Identification of dosage-sensitive genes in Saccharomyces cerevisiae using the genetic tug-of-war method Genome Res 2013 23 300 311 10.1101/gr.146662.112 23275495 PMC3561871

[B12] Giaever G Chu AM Ni L Connelly C Riles L Véronneau S Dow S Lucau-Danila A Anderson K André B Functional profiling of the Saccharomyces cerevisiae genome Nature 2002 418 387 391 10.1038/nature00935 12140549

[B13] Costanzo M Baryshnikova A Bellay J Kim Y Spear ED Sevier CS Ding H Koh JL Toufighi K Mostafavi S The genetic landscape of a cell Science 2010 327 425 431 10.1126/science.1180823 20093466 PMC5600254

[B14] Hillenmeyer ME Ericson E Davis RW Nislow C Koller D Giaever G Systematic analysis of genome-wide fitness data in yeast reveals novel gene function and drug action Genome Biol 2010 11 R30 10.1186/gb-2010-11-3-r30 20226027 PMC2864570

[B15] Mnaimneh S Davierwala AP Haynes J Moffat J Peng WT Zhang W Yang X Pootoolal J Chua G Lopez A Exploration of essential gene functions via titratable promoter alleles Cell 2004 118 31 44 10.1016/j.cell.2004.06.013 15242642

[B16] Drinnenberg IA Weinberg DE Xie KT Mower JP Wolfe KH Fink GR Bartel DP RNAi in budding yeast Science 2009 326 544 550 10.1126/science.1176945 19745116 PMC3786161

[B17] Breslow DK Cameron DM Collins SR Schuldiner M Stewart-Ornstein J Newman HW Braun S Madhani HD Krogan NJ Weissman JS A comprehensive strategy enabling high-resolution functional analysis of the yeast genome Nat Methods 2008 5 711 718 10.1038/nmeth.1234 18622397 PMC2756093

[B18] Schuldiner M Collins SR Thompson NJ Denic V Bhamidipati A Punna T Ihmels J Andrews B Boone C Greenblatt JF Exploration of the function and organization of the yeast early secretory pathway through an epistatic miniarray profile Cell 2005 123 507 519 10.1016/j.cell.2005.08.031 16269340

[B19] Finoux AL Séraphin B In vivo targeting of the yeast Pop2 deadenylase subunit to reporter transcripts induces their rapid degradation and generates new decay intermediates J Biol Chem 2006 281 25940 25947 10.1074/jbc.M600132200 16793769

[B20] Kanemaki M Sanchez-Diaz A Gambus A Labib K Functional proteomic identification of DNA replication proteins by induced proteolysis in vivo Nature 2003 423 720 724 10.1038/nature01692 12768207

[B21] Nishimura K Fukagawa T Takisawa H Kakimoto T Kanemaki M An auxin-based degron system for the rapid depletion of proteins in nonplant cells Nat Methods 2009 6 917 922 10.1038/nmeth.1401 19915560

[B22] Taxis C Stier G Spadaccini R Knop M Efficient protein depletion by genetically controlled deprotection of a dormant N-degron Mol Syst Biol 2009 5 267 19401679 10.1038/msb.2009.25 PMC2683728

[B23] Taxis C Knop M TIPI: TEV protease-mediated induction of protein instability Methods Mol Biol 2012 832 611 626 10.1007/978-1-61779-474-2_43 22350916

[B24] Varshavsky A The N-end rule pathway of protein degradation Genes Cells 1997 2 13 28 10.1046/j.1365-2443.1997.1020301.x 9112437

[B25] Ugolini S Bruschi CV The red/white colony color assay in the yeast Saccharomyces cerevisiae: epistatic growth advantage of white ade8-18, ade2 cells over red ade2 cells Curr Genet 1996 30 485 492 10.1007/s002940050160 8939809

[B26] Stevenson LF Kennedy BK Harlow E A large-scale overexpression screen in Saccharomyces cerevisiae identifies previously uncharacterized cell cycle genes Proc Natl Acad Sci USA 2001 98 3946 3951 10.1073/pnas.051013498 11274415 PMC31159

[B27] Tavormina PA Burke DJ Cell cycle arrest in cdc20 mutants of Saccharomyces cerevisiae is independent of Ndc10p and kinetochore function but requires a subset of spindle checkpoint genes Genetics 1998 148 1701 1713 9560388 10.1093/genetics/148.4.1701 PMC1460108

[B28] Lin YY Qi Y Lu JY Pan X Yuan DS Zhao Y Bader JS Boeke JD A comprehensive synthetic genetic interaction network governing yeast histone acetylation and deacetylation Genes Dev 2008 22 2062 2074 10.1101/gad.1679508 18676811 PMC2492751

[B29] Schott EJ Hoyt MA Dominant alleles of Saccharomyces cerevisiae CDC20 reveal its role in promoting anaphase Genetics 1998 148 599 610 9504909 10.1093/genetics/148.2.599 PMC1459839

[B30] Tsuchiya D Gonzalez C Lacefield S The spindle checkpoint protein Mad2 regulates APC/C activity during prometaphase and metaphase of meiosis I in Saccharomyces cerevisiae Mol Biol Cell 2011 22 2848 2861 10.1091/mbc.E11-04-0378 21697504 PMC3154881

[B31] Lew DJ Weinert T Pringle JR Pringle JR, Broach JR, Jones EW Cell cycle control in Saccharomyces cerevisiae The Molecular and Cellular Biology of the Yeast Saccharomyces: Cell Cycle and Cell Biology 1997 Cold Spring Harbor, NY: Cold Spring Harbor Laboratory Press 607 695

[B32] Bandyopadhyay S Mehta M Kuo D Sung MK Chuang R Jaehnig EJ Bodenmiller B Licon K Copeland W Shales M Rewiring of genetic networks in response to DNA damage Science 2010 330 1385 1389 10.1126/science.1195618 21127252 PMC3006187

[B33] Chen KC Calzone L Csikasz-Nagy A Cross FR Novak B Tyson JJ Integrative analysis of cell cycle control in budding yeast Mol Biol Cell 2004 15 3841 3862 10.1091/mbc.E03-11-0794 15169868 PMC491841

[B34] Baryshnikova A Costanzo M Dixon S Vizeacoumar FJ Myers CL Andrews B Boone C Synthetic genetic array (SGA) analysis in Saccharomyces cerevisiae and Schizosaccharomyces pombe Methods Enzymol 2010 470 145 179 20946810 10.1016/S0076-6879(10)70007-0

[B35] Baryshnikova A Costanzo M Kim Y Ding H Koh J Toufighi K Youn JY Ou J San Luis BJ Bandyopadhyay S Quantitative analysis of fitness and genetic interactions in yeast on a genome scale Nat Methods 2010 7 1017 1024 10.1038/nmeth.1534 21076421 PMC3117325

[B36] Li Z Vizeacoumar FJ Bahr S Li J Warringer J Vizeacoumar FS Min R Vandersluis B Bellay J Devit M Systematic exploration of essential yeast gene function with temperature-sensitive mutants Nat Biotechnol 2011 29 361 367 10.1038/nbt.1832 21441928 PMC3286520

[B37] Deutschbauer AM Jaramillo DF Proctor M Kumm J Hillenmeyer ME Davis RW Nislow C Giaever G Mechanisms of haploinsufficiency revealed by genome-wide profiling in yeast Genetics 2005 169 1915 1925 10.1534/genetics.104.036871 15716499 PMC1449596

[B38] Springer M Weissman JS Kirschner MW A general lack of compensation for gene dosage in yeast Mol Syst Biol 2010 6 368 20461075 10.1038/msb.2010.19 PMC2890323

[B39] Brachmann CB Davies A Cost GJ Caputo E Li J Hieter P Boeke JD Designer deletion strains derived from Saccharomyces cerevisiae S288C: a useful set of strains and plasmids for PCR-mediated gene disruption and other applications Yeast 1998 14 115 132 10.1002/(SICI)1097-0061(19980130)14:2<115::AID-YEA204>3.0.CO;2-2 9483801

[B40] Amberg DC Burke D Strathern JN Methods in Yeast Genetics: A Cold Spring Harbor Laboratory Course Manual 2005 New York: Cold Spring Harbor Laboratory Press

[B41] Goldstein AL McCusker JH Three new dominant drug resistance cassettes for gene disruption in Saccharomyces cerevisiae Yeast 1999 15 1541 1553 10.1002/(SICI)1097-0061(199910)15:14<1541::AID-YEA476>3.0.CO;2-K 10514571

