# Supplementary material for: Evaluation of the lower protein limit in the budding yeast Saccharomyces cerevisiae using TIPI-gTOW
Source: BMC Syst Biol. 2014 Jan 7;8:2. doi: 10.1186/1752-0509-8-2 (PMC3892067; doi:10.1186/1752-0509-8-2)
Supplement: Additional file 1 — This file contains 3 tables (Table S1. Summary of construction of GFP-DegF integrated strains with different lengths of CDC19 promoters. Table S2. Upper limits of the rates of degradation of Cdc20 (kd20) in gene-deletion models. Table S3. PCR primers used in this study.) and 4 figures (Figure S1. Serial deletions of the CDC19 promoter. Figure S2. Correlation between the copy numbers of vector and TEV plasmids. Figure S3. Potential outcomes of TIPI-gTOW of Cdc20 in cell cycle regulator deletion strains and their regulatory interactions (Case 1). Figure S4. Potential outcomes of TIPI-gTOW of Cdc20 in cell cycle regulator deletion strains and their regulatory interactions (Case 2)). [file 1752-0509-8-2-S1.docx]

Supplementary Information for “**Evaluation of the lower protein limit in the budding yeast *Saccharomyces cerevisiae* using TIPI-gTOW**” by Masataka Sasabe *et al*.

# Table of contents

Supplementary Tables 2

**Table S1.** Summary of construction of *GFP-DegF* integrated strains with different lengths of *CDC19* promoters 2

**Table S2**. Upper limits of the rates of degradation of Cdc20 (kd20) in gene-deletion models 3

**Table S3**. PCR primers used in this study 4

Supplementary Figures 5

**Figure S1**. Serial deletions of the *CDC19* promoter 5

**Figure S2**. Correlation between the copy numbers of vector and TEV plasmids 6

**Figure S3**. Potential outcomes of TIPI-gTOW of Cdc20 in cell cycle regulator deletion strains and their regulatory interactions (Case 1) 7

**Figure S4**. Potential outcomes of TIPI-gTOW of Cdc20 in cell cycle regulator deletion strains and their regulatory interactions (Case 2) 8

# Supplementary Tables

## **Table S1.** Summary of construction of *GFP-DegF* integrated strains with different lengths of *CDC19* promoters

|  | *CDC19* promoter | *CDC19_-600_* promoter | *CDC19_-500_* promoter |
| --- | --- | --- | --- |
| *CDC15* | ○ | ○ | ○  (YSM002) |
| *CDC20* | × | × | ○*  (YSM003) |
| *CDC28* | ○ | ○*  (YSM005) | △  (YSM004) |

○: integrated strain obtained, ×: integrated strain not obtained, △: integrated strain obtained but transformant with the TEV plasmid not obtained, *: significant reduction in the copy number of the TEV plasmid observed. Strain names used in Figure 3 are also shown.

## **Table S2**. Upper limits of the rates of degradation of Cdc20 (kd20) in gene-deletion models

|  | Upper limit kd20 | Relative change |
| --- | --- | --- |
| Wild type | 0.58 | 0.00 |
| *clb2Δ* | 0.52 | ­­−0.09 |
| *lteΔ* | 0.59 | 0.01 |
| *mcmΔ* | 0.31 | −0.46 |
| *cdhΔ* | 0.16 | −0.73 |
| *sicΔ* | 0.89 | 0.54 |
| *sbfΔ* | 0.85 | 0.47 |
| *mbfΔ* | 0.16 | −0.72 |
| *cln2Δ* | 0.87 | 0.50 |
| *clb5Δ* | 0.48 | −0.18 |
| *swi5*Δ | 1.41 | 1.45 |
| *bck2Δ* | 0.92 | 0.59 |
| *bub2Δ* | 0.59 | 0.01 |
| *mad2Δ* | 1.17 | 1.03 |

## **Table S3**. PCR primers used in this study

| Name | Sequence (5′ to 3′) |
| --- | --- |
| OHML432 | ccttttattaagaactaaatggacaatattatggagcattcggccgctctagaactagtG |
| OHML389 | attgggtaccgggccccccCTCGAGAACTACAAAGATATCCTGAC |
| OHML504 | TCCACCTTGCGATGGTCTTTCCGCTTTTCTTGCTGTTATTTTTGTATACGgtccccgccgggtcacccgg |
| OHML505 | GCCCTCTGGATGGGAGTCAAGTTGACTCTATCGGTATCGGCCATACTGTTtactgcaggaattcgatcca |
| OHML506 | TAAACCAGAGATCAGTCATCAAGCTTAGGACTAATCTGCTTTGCGACTTTgtccccgccgggtcacccgg |
| OHML507 | GAACGGTTACCGCTAATTGCTGCATTTCCCTTATCTCTAGAGCTTTCTGGtactgcaggaattcgatcca |
| OHML508 | AAAAAAAAAAATTGGTGGAAGGACCAAGTCCTCTTGAAAGAAAATTTAATgtccccgccgggtcacccgg |
| OHML509 | GTACCTTCACCGACTTTCTCAAGTCTTTTGTAATTTGCTAATTCACCGCTtactgcaggaattcgatcca |
| OHML541 | tgtcgattcgatactaacgccgccatccagAAAAGGAAAGATTATTGAAA |
| OHML542 | tgtcgattcgatactaacgccgccatccagAACGGCGGGATTCCTCTATG |
| OHML543 | tgtcgattcgatactaacgccgccatccagACAGATTGGGAGATTTTCAT |
| OHML544 | tgtcgattcgatactaacgccgccatccagCATTCAGTTGAGTTGAGTGA |
| OHML545 | tgtcgattcgatactaacgccgccatccagTTCCTTTCCTTCCCATATGA |
| OHML546 | tgtcgattcgatactaacgccgccatccagTTATAAATACTCTTTGGTAA |
| OHML547 | tgtcgattcgatactaacgccgccatccagATTCTTTTTCATCCTTTGGT |
| OHML540 | Ctggatggcggcgttagtat |
| LEU2-2F | GCTAATGTTTTGGCCTCTTC |
| LEU2-2R | ATTTAGGTGGGTTGGGTTCT |
| LEU3-3F | CAGCAACTAAGGACAAGG |
| LEU3-3R | GGTCGTTAATGAGCTTCC |

# Supplementary Figures


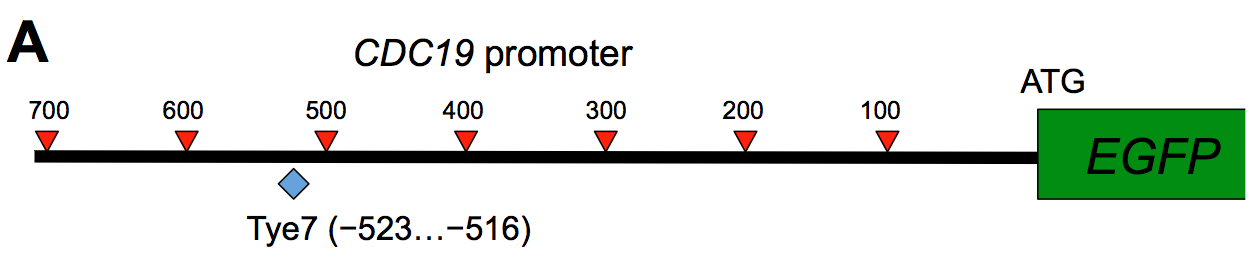
**
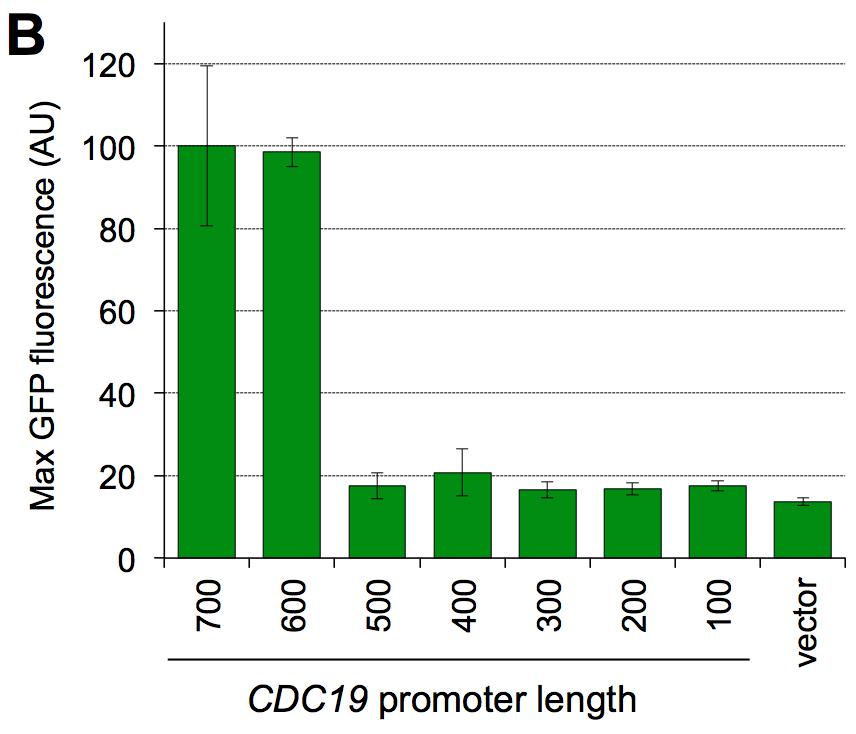

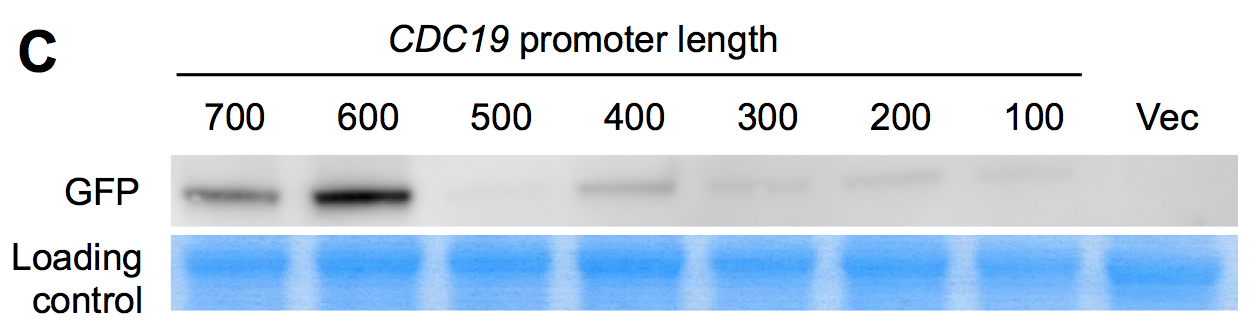
**

## **Figure S1**. Serial deletions of the *CDC19* promoter

(**A**) A map of the *CDC19* promoter. The number corresponds to the nucleotide number from the start codon. The Tye7 binding site between –600 bp to –500 bp is shown. (**B**) Maximum GFP fluorescence expressed from the GFP gene with *CDC19* promoters of indicated sizes. Yeast BY4741 cells harboring pSS1002 derivatives with truncated *CDC19* promoters or the empty vector (pRS423ks) were cultivated in SC–His medium in a microtiter plate at 30˚C without shaking, and the GFP fluorescence was measured using Infinite F200 microplate reader (Tecan) with the GFP filter (485/540nm) every 30 min for 50 h. Four independent measurements were performed, and the average is shown. The error bar indicates the standard deviation. All cells harboring pSS1002 derivatives (100 bp to 700 bp) showed significantly higher GFP fluorescence than the vector control (*p* < 0.05 in Student’s one tail t-test). (**C**) Western blotting of GFP expressed from *CDC19* promoters of various lengths. Yeast BY4741 cells harboring pSS1002 derivatives with truncated *CDC19* promoters were cultivated in SC–His medium at 30˚C until the mid-log phase. Western blotting was performed as described in Methods of the main text. As loading controls, Coomassie^®^ G-250 staining of 50 kDa bands (corresponding to the size of EF-1α) are shown. The intensities of the GFP bands showed a good correlation with the GFP fluorescence shown in **B** (Pearson’s correlation coefficient = 0.90).


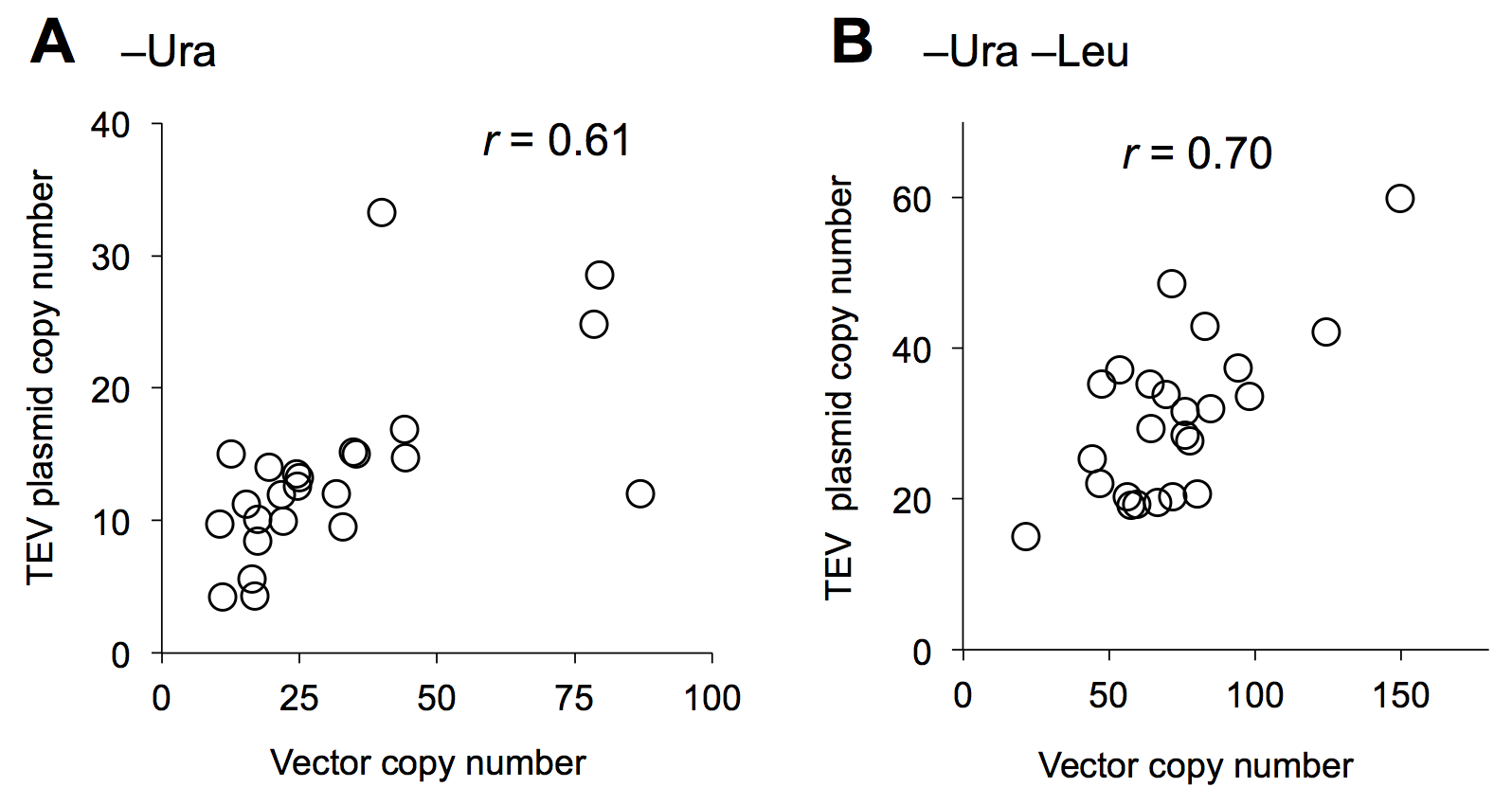


## **Figure S2**. Correlation between the copy numbers of vector and TEV plasmids

Scatter plots of the copy numbers of vector and TEV plasmids under the –Ura condition (**A**) and –Ura−Leu condition (**B**). Pearson’s correlation coefficient (*r*) for each condition is shown.

**
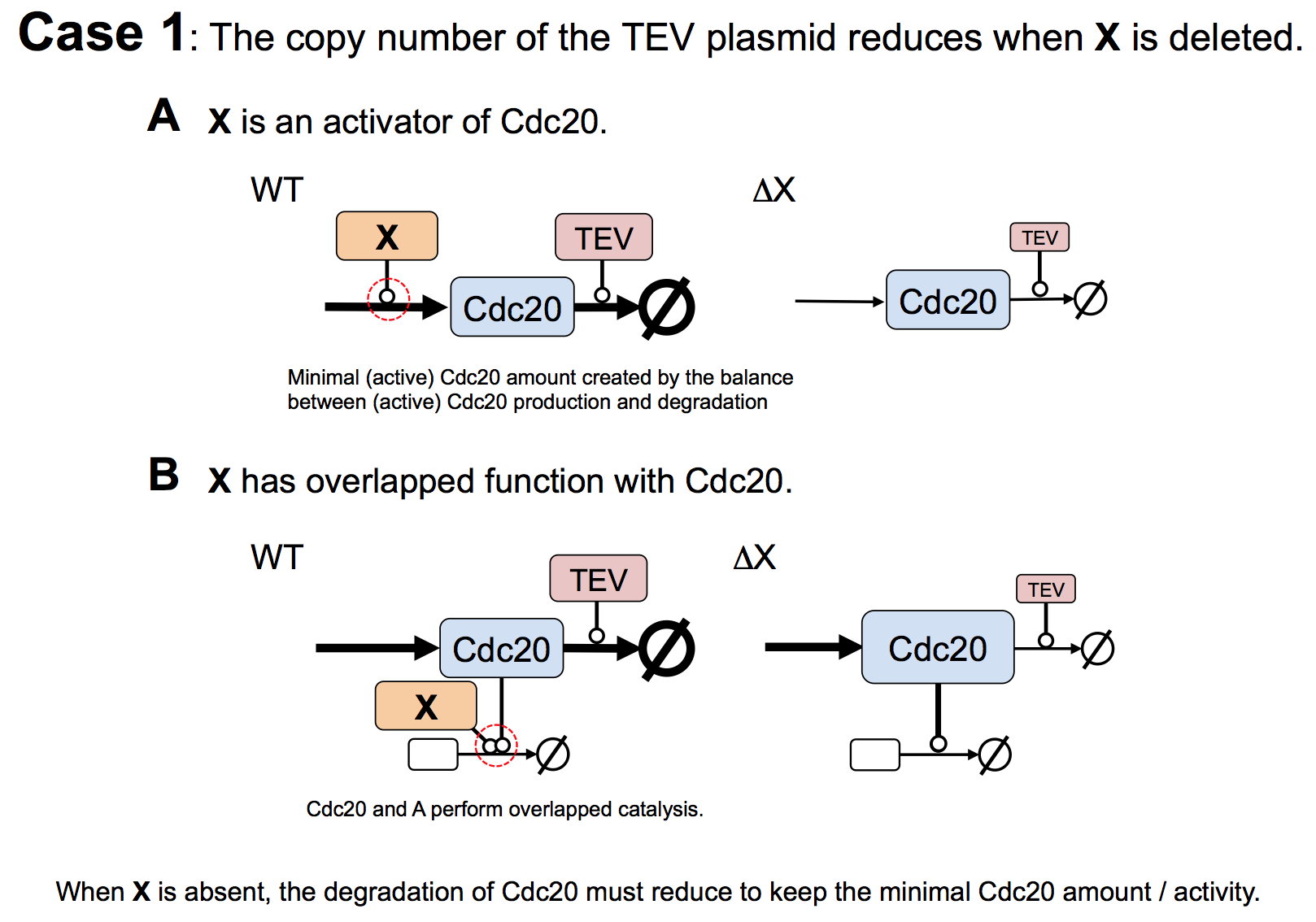
**

## **Figure S3**. Potential outcomes of TIPI-gTOW of Cdc20 in cell cycle regulator deletion strains and their regulatory interactions (Case 1)

**Case 1**: **A** and **B** are potential regulatory pathways if the copy number of the TEV plasmid reduces when the cell cycle regulator X is deleted. (**A**) The case in which X is an activator of Cdc20. (**B**) The case in which X has a function overlapping with that of Cdc20. In both cases, the degradation of Cdc20 must be reduced to maintain the minimal level of Cdc20 or activity. The diagrams were drawn using SBGN (www.sbgn.org).


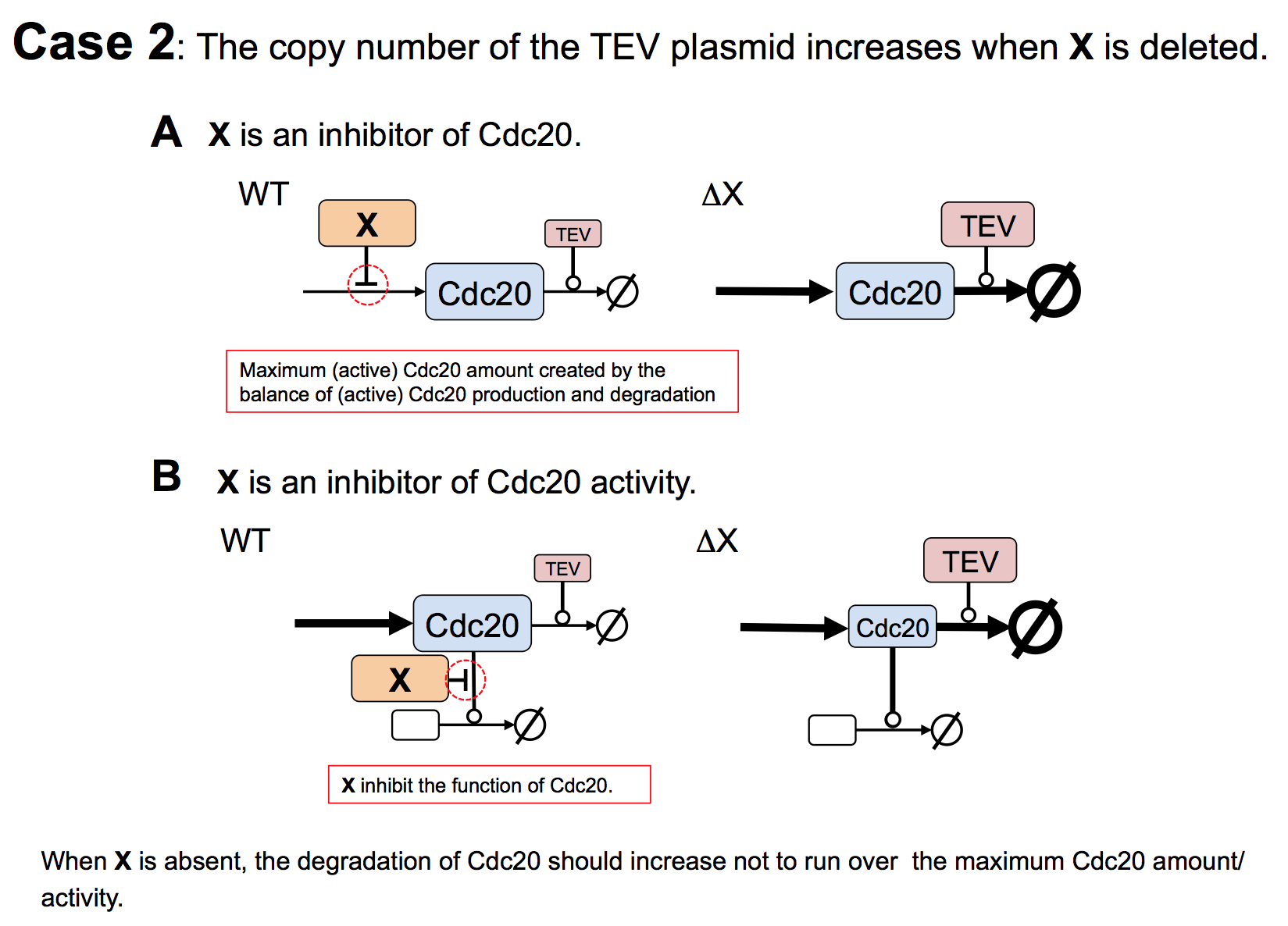


## **Figure S4**. Potential outcomes of TIPI-gTOW of Cdc20 in cell cycle regulator deletion strains and their regulatory interactions (Case 2)

**Case 2**: **A** and **B** are potential regulatory pathways if the copy number of the TEV plasmid increases when cell cycle regulator X is deleted. (**A**) The case in which X is an inhibitor of Cdc20. (**B**) The case in which X is an inhibitor of Cdc20 activity. In both cases, the degradation of Cdc20 should increase so that the maximum Cdc20 amount or activity is not exceeded. The diagrams were drawn using SBGN (www.sbgn.org).
